# Pilot study in human healthy volunteers on the use of magnetohydrodynamics in needle-free continuous glucose monitoring

**DOI:** 10.1038/s41598-022-21424-9

**Published:** 2022-11-09

**Authors:** Tuuli A. Hakala, Laura K. Zschaechner, Risto T. Vänskä, Teemu A. Nurminen, Melissa Wardale, Jonathan Morina, Zhanna A. Boeva, Reeta Saukkonen, Juha-Matti Alakoskela, Kim Pettersson-Fernholm, Edward Hæggström, Johan Bobacka, Alejandro García Pérez

**Affiliations:** 1 Glucomodicum Ltd, A.I. Virtasen Aukio 1, 00560 Helsinki, Finland; 2 Laboratory of Molecular Science and Engineering, Faculty of Science and Engineering, Åbo Akademi University, Biskopsgatan 8, 20500 Turku/Åbo, Finland; 3 Skin and Allergy Hospital, Meilahdentie 2, 00250 Helsinki, Finland; 4 Nefrologian Poliklinikka, Helsinki University Hospital, Haartmaninkatu 4, 00029 Helsinki, Finland; 5 Department of Physics, University of Helsinki, Gustaf Hällströmin katu 2, 00560 Helsinki, Finland

**Keywords:** Biophysics, Biomarkers, Physics

## Abstract

The benefits of continuous glucose monitoring (CGM) in diabetes management are extensively documented. Yet, the broader adoption of CGM systems is limited by their cost and invasiveness. Current CGM devices, requiring implantation or the use of hypodermic needles, fail to offer a convenient solution. We have demonstrated that magnetohydrodynamics (MHD) is effective at extracting dermal interstitial fluid (ISF) containing glucose, without the use of needles. Here we present the first study of ISF sampling with MHD for glucose monitoring in humans. We conducted 10 glucose tolerance tests on 5 healthy volunteers and obtained a significant correlation between the concentration of glucose in ISF samples extracted with MHD and capillary blood glucose samples. Upon calibration and time lag removal, the data indicate a Mean Absolute Relative Difference (MARD) of 12.9% and Precision Absolute Relative Difference of 13.1%. In view of these results, we discuss the potential value and limitations of MHD in needle-free glucose monitoring.

## Introduction

Glucose monitoring has a fundamental role in the prevention and management of diabetes, the fastest growing disease in the world^1^. Nearly 10% of the world’s population, living with diabetes, rely on periodic glucose monitoring to manage the disease. Traditional capillary blood glucose (CBG) meters measure the concentration of glucose from capillary blood samples obtained by the patient. Each measurement requires pricking a finger with a lancet to obtain a blood sample, a painful and inconvenient procedure.

Current trends lean towards continuous glucose monitoring (CGM). Novel devices based on interstitial fluid (ISF) include Freestyle Libre from Abbot^2^, Dexcom G6 from Dexcom^3^ (subcutaneous needle) and Eversense from Senseonics^4^ (subcutaneous sensor). These devices improve the quality of life for people living with diabetes^5^, and effectively, help patients to better manage the disease. For instance, persons with type-1 diabetes who use CGM devices are less prone to hypoglycemia^6,7^, stay longer within target glucose range^8,9^, and have reduced HbA1c levels^6–9^. Similar results have been observed in patients with type 2 diabetes^10,11^. Devices for CGM enable glucose trend tracking, which has sparked interest beyond diagnosed cases of diabetes. For example, CGM can motivate people with pre-diabetes to make improvements in life-style choices. It can also aid in optimizing the recovery and peak performance of athletes through nutrition management^12,13^.

While traditional glucose meters use capillary blood, most commercial CGM devices measure glucose from ISF, which can be accessed using minimally invasive or non-invasive approaches. Many compounds, such as glucose, are transported from the blood into the cells via ISF^14,15^. The glucose concentration in ISF strongly correlates with the blood glucose concentration^16,17^. This correlation makes ISF attractive for glucose monitoring. However, sampling of ISF non-invasively is challenging due to the barrier function of the skin. Thus, all approaches based on ISF that have reached commercial success are based on needles or microneedles that penetrate the skin and reach the ISF in the dermis.

Other approaches for non-invasive CGM under active research include sampling of interstitial fluid with reverse iontophoresis^18–20^ and ultrasound^21,22^, and detection of glucose through the skin using light^23^, or radio waves^24^. These approaches, however, are yet to demonstrate their capability of delivering the required accuracy and precision out of laboratory settings.

Both invasive and non-invasive methods for CGM in interstitial fluid face the common challenge of a lag time between the concentrations of glucose in ISF and in blood. The glucose concentration in ISF depends on the diffusion rate of glucose from capillaries to the interstitial fluid and the rate of glucose intake by cells^25^. Consequently, most of the time, the glucose concentration in ISF lags the concentration of glucose in blood^26–28^. The magnitude of this lag varies with time^26^ and within a person over time depending on their physical activity and blood glucose concentrations^27^.

The changing lag time increases the complexity of determining the accuracy (proximity to the true value) and precision (spread) of individual glucose measurements on ISF using blood glucose measurements as the reference. The accuracy of glucose monitoring methods is typically quantified using the Mean Absolute Relative Difference (MARD) and, with increasing popularity, also the Precision Absolute Relative difference (PARD)^29^. Error grids such as the Consensus (i.e., Parkes) Error Grid (CEG)^30^ visually represent the accuracy. These error grids display predicted vs. reference blood glucose concentrations and are divided into risk zones A-E that correspond to the severity of clinical outcome associated with a discrepancy between the predicted and reference glucose^31^.

In our previous work^32^, we introduced MHD as a novel physical mechanism for extraction of dermal interstitial fluid. Also, using an ex vivo model, we demonstrated a 13-fold increase in extraction rate of glucose when a 300 mT magnetic field is added^32^. In this paper we present for the first time the use of MHD for extraction of interstitial fluid in healthy volunteers and its application to glucose monitoring. Over the course of a glucose tolerance test, we obtain samples of interstitial fluid with MHD (needle-free) from the skin of healthy volunteers. Concurrently, we measure CBG with a reference glucose meter. We determine the glucose concentration in the ISF samples using a fluorometric assay. Then, we investigate the correlation between the two sets of measurements. To improve the reliability of the correlation analysis, we calibrate the ISF measurements using a least squares optimization algorithm to consider the temporal lag.

## Results

### Sampling of interstitial fluid using MHD

**Figure 1:**
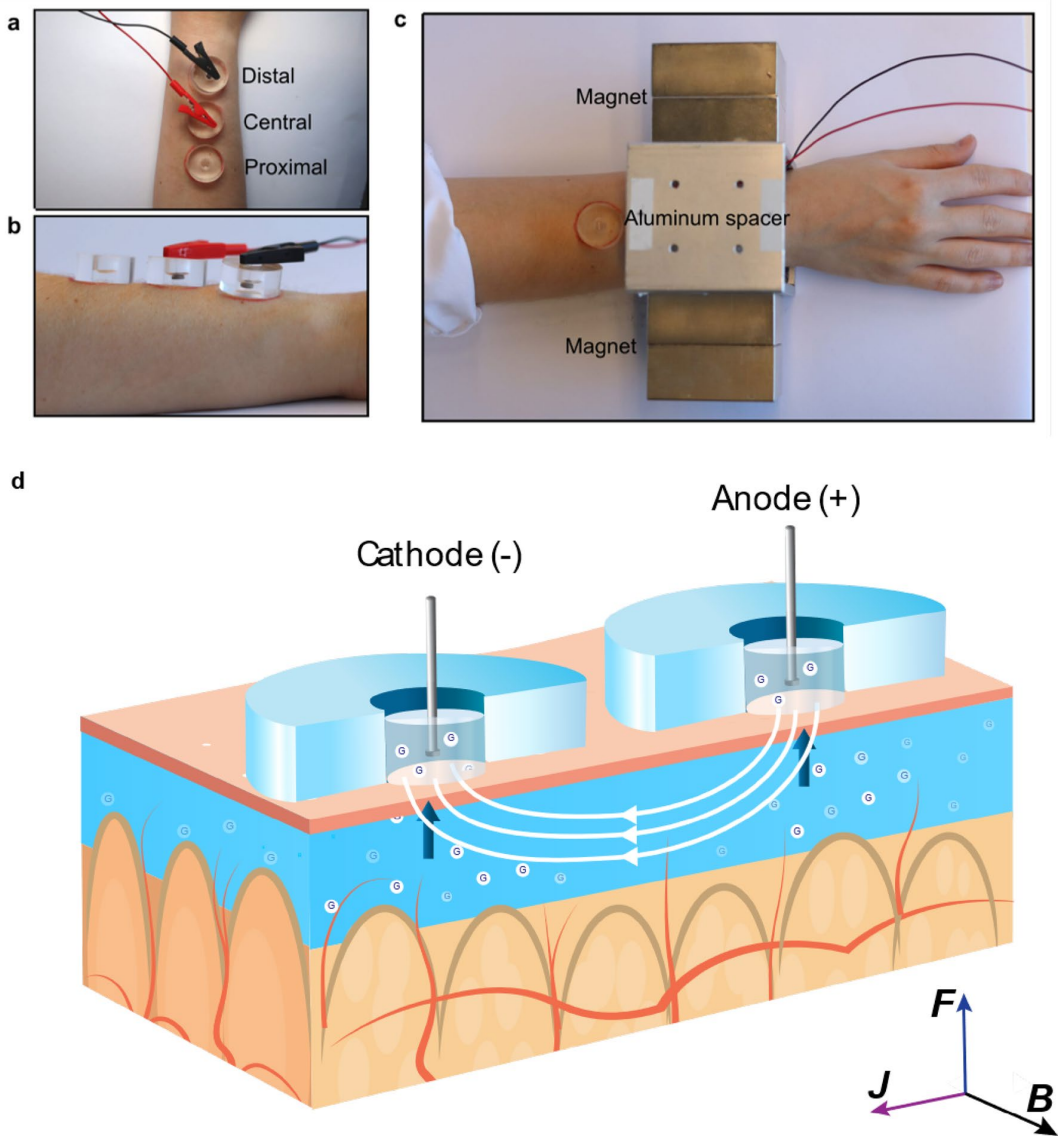
Experimental set up and scheme. (**a**) Top-view of the acrylic wells placed on the volunteer’s lower arm. (**b**) A side-view of the acrylic wells. (**c**) The volunteer’s arm was placed between two neodymium magnets separated by an aluminum support. (** d**) A schematic presentation of the set-up and the MHD extraction. Two Ag/AgCl disk electrodes inside the well drive a current through the skin (***J***). A magnetic field with direction (***B***) orthogonal to the current is applied to create Lorentz force (***F***) pointing towards the skin surface. The scheme is not drawn to scale. Figure Credits: Artic Frame Studio https://www.arcticframe.com.

We developed a setup for ISF sampling, consisting of acrylic wells, electrodes, and block magnets (Fig. 1a-d). The position of the wells mimics the position of a fitness watch, and we positioned them on the flattest part of the dorsal side of the lower arm.

### MHD extraction during glucose tolerance tests

**Figure 2:**
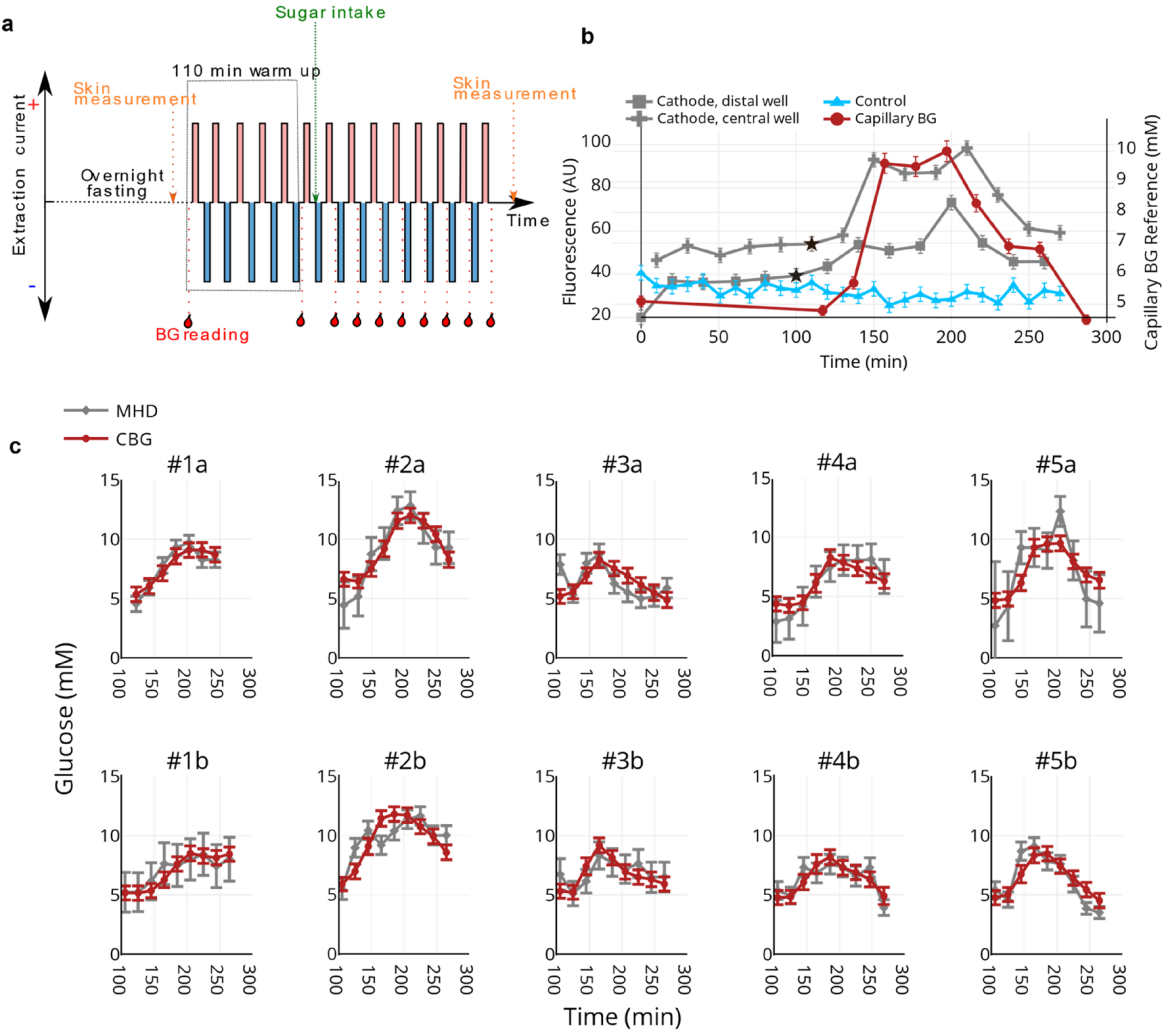
Glucose tolerance tests. (** a**) Schematic presentation of the glucose tolerance test timeline (black dashed line)**.** Volunteers entered the test after overnight fasting (9 h). 5 min MHD extractions were conducted with alternating current polarity. The red and blue bars indicate the direction of the applied electric current for each extraction. Capillary blood glucose measurements, represented by red blood drops and red dashed lines, were taken twice before sugar intake (green dashed line). 8 more capillary blood glucose readings were obtained after the glucose drink with 20 min intervals. The skin hydration and transepidermal water loss readings were recorded before and after the test. (**b**) Raw data from a representative experiment. These data are from the MHD cathode central well (grey diamonds), the control well (blue triangles), and reference blood glucose measurements (red circles). The first data point after warm-up is shown as a star. Error bars for the MHD and diffusion raw data are based on the standard error of the diffusion data recorded after the warm-up period. Error bars for the CBG concentrations are based on the standard error of our reference device. (**c**) All 10 experiments shown after linear interpolation of the reference CBG and after applying calibration. Each column consists of two experiments involving a single participant. Error estimates for the interstitial fluid glucose (ISFG) (not shown) are based on a combination of the standard error of the control well measurements and the standard error of our reference device and are in almost all cases ± 1–2 mM.

The performance of MHD extraction was evaluated by conducting a series of glucose tolerance tests. Figure 2a shows a schematic of the timeline for an individual glucose tolerance test.

The extraction voltages drifted during the first ~ 100 min of each experiment (warm-up period). Thus, MHD samples extracted prior to 100 min were not used to investigate the correlation between ISFG and CBG concentrations.

We used an enzymatic assay to determine the amount of glucose in the extracted samples. Figure 2b). MHD and CBG glucose readings were taken from both the left (n = 5, Fig. 2c #1a-#5a) and the right (n = 5, Fig. 2c #1b-#5b) arms. The results from all 10 experiments after least squares-based calibration, as described in “Materials and Methods”, are presented in Fig. 2c. During the 300 min of sampling, we obtained a blood glucose peak for each volunteer. In most cases, the same individual exhibited a similar profile for the glucose tolerance test in the two experiment days. Volunteer #1, for instance, had a slow rise of CBG reading and only slight drop after the peak at 200 min before the experiment was concluded on the two experiment days (#1a and #1b).

### Correlation between glucose in extracted ISF samples and blood glucose concentrations

**Figure 3:**
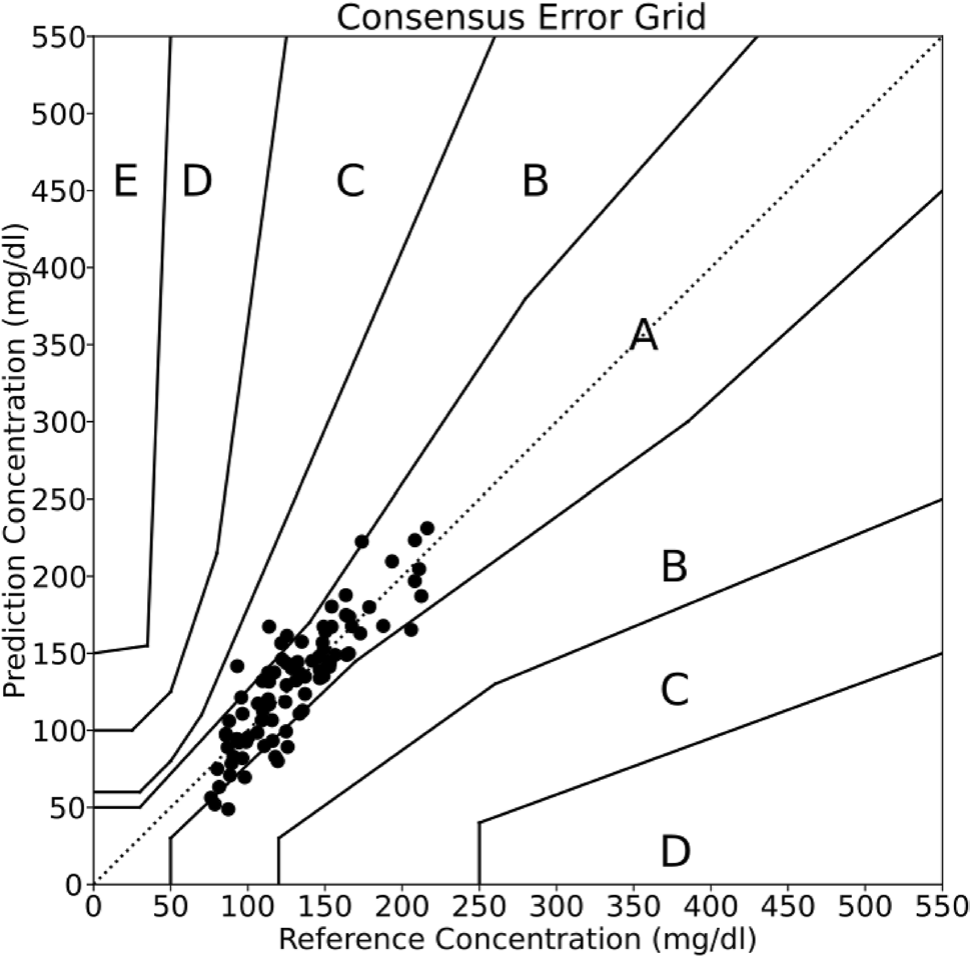
Correlation of glucose concentrations in extracted ISF and capillary blood samples. CEG showing 88 measurement pairs from 10 experiments. These data are shown after applying the basic least squares calibration algorithm. Percentages within each zone are as follows: Zone A: 84.0%, Zone B: 16.0%, Zones C-E: 0%. The corresponding MARD is 12.9%.

The results from the glucose tolerance tests are shown in a CEG (Fig. 3). In total, the ten experiments yielded 88 measurement pairs. Of these measurement pairs, 84% fell within Zone A and 16% fell within Zone B. The corresponding MARD value is 12.9%. We found a correlation (R^2^ = 0.92) between the MHD extracted glucose concentrations and the reference blood glucose measurements. The resulting relationship between MHD glucose concentrations and reference CBG indicates the presence of a temporal lag between CBG and ISFG. Such a lag has also been observed by others^17,27,28,33,34^, and was thus expected. We accounted for this lag in our calibration (described in Materials and Methods).

### Effect of extraction on the skin

**Figure 4:**
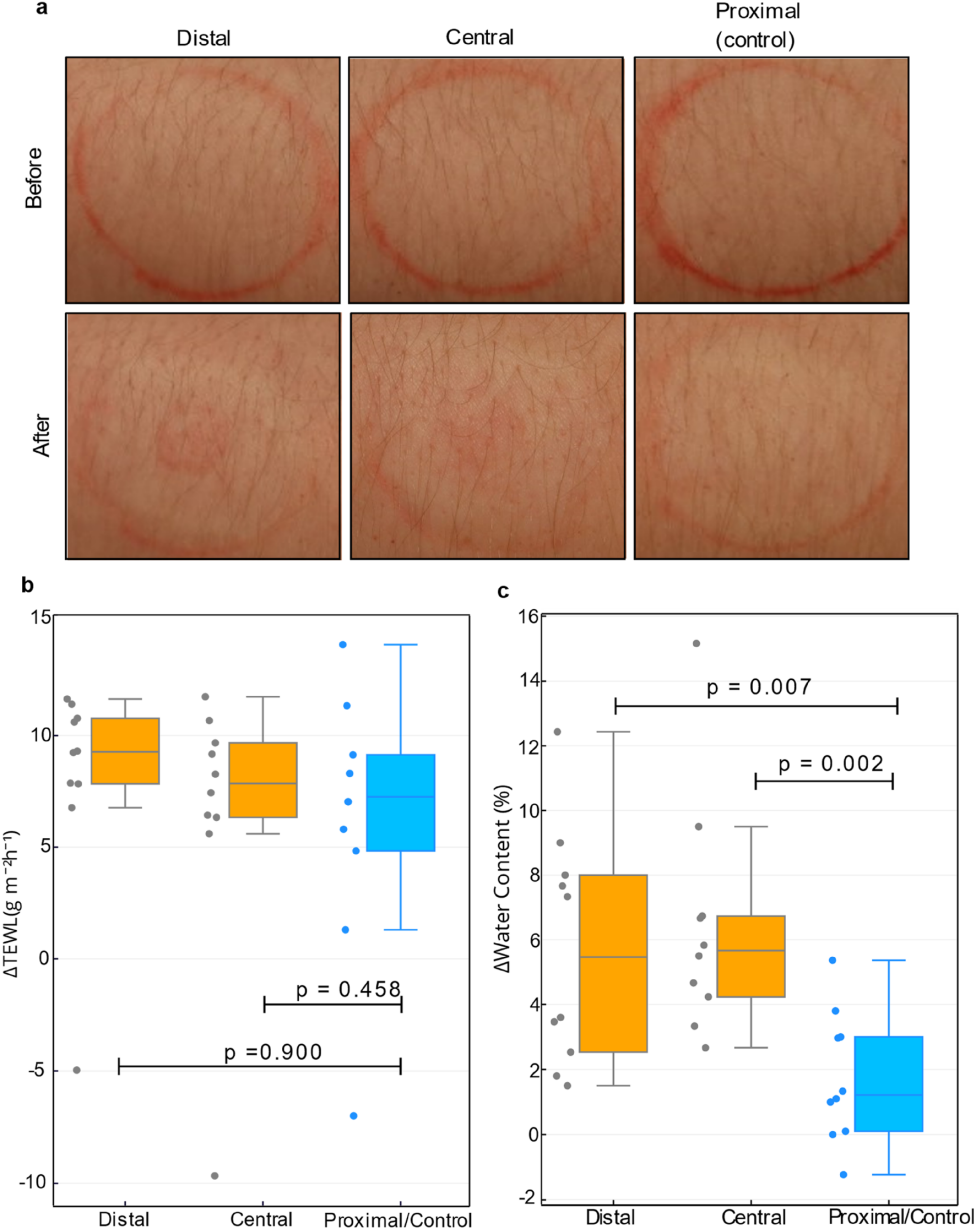
Effect of extraction on the skin. (**a**) Photographs of the skin MHD well sites before and after the glucose tolerance test that includes 30 extractions lasting 5 min. The extraction well outline was marked with a red makeup pen before the experiment. (**b**) ΔTEWL and (**c**) Δ skin water content for each well from all 10 experiments. Δ values were obtained by measuring the skin parameters before and after the experiment and by calculating the difference between these values (Δ = after – before). *p* values were obtained via t-test, with *p* ≤ 0.05 considered significant.

To investigate potential effects of MHD extraction on the skin, we took photographs of the skin (Fig. 4a) and measured the transepidermal water loss (TEWL) before and after the glucose tolerance test. Thus, we examined the effect of multiple extractions. The skin photographs were visually analysed by an experienced dermatologist. We observed slight redness under extraction wells (distal and central wells) immediately after the experiment for all participants. This redness disappeared after a few hours. In some cases, the adhesive caused slight temporary redness on the participant’s arm. Figure 4b shows that the TEWL rate changed due to the extraction experiment on the volar forearm locations of the two active wells and the control well. Figure S3a, shows the TEWL measured at the extraction sites before and after the extraction experiment. The results demonstrate a higher TEWL at the more distal location on the forearm, which is consistent with the reported values and demonstrate a known trend for higher TEWL on a more distal part of the forearm^35^. Although the TEWL rate increased in measurements done 15 min after the test, a comparable increase was also observed for the control well, implying that this observation mostly resulted from the skin occlusion, a known cause of a transient increase in TEWL. Occlusion is occasionally used as a skin barrier stress test in combination with TEWL measurements^36^. Furthermore, we observed increased epidermal and dermal water content after the extraction experiments (Fig. 4c), with a greater increase at the MHD active well locations (distal and central) compared to the control well location (proximal). The *p*-test of epidermal and dermal water content in MHD wells compared to the control well resulted in statistically significant difference of *p* = 0.007 and *p* = 0.002, respectively.

## Discussion

While ongoing research efforts target non-invasive solutions to CGM, such solutions have so far been elusive. Over the last few years, CGM devices based on subcutaneous needles have developed rapidly to meet high standards for accuracy and safety^1^. These technologies benefit a rapidly growing number of people with diabetes, in particular, insulin users^37^. However, the adoption of CGM devices by people with- or at risk of getting diabetes remains low^38^. To solve this problem, further development of existing and new technologies for CGM should lead towards reducing costs, improving usability, and minimizing environmental impact. A clear path towards improvement on these 3 areas is the development of a non-invasive CGM solution. For instance, a solution based on extraction of interstitial fluid with MHD can be implemented in the form of a reusable wearable device combined with disposable low-cost screen-printed biosensors.

In this study we present needle-free ISF glucose sampling using MHD in healthy volunteers. We performed 10 glucose tolerance tests on 5 healthy volunteers while sampling CBG concentrations and ISFG concentrations (Fig. 2). The resulting data and calibration yielded a MARD of 12.9% and a PARD of 13.1%. These values are within the range 10–15% typically reported by commercial CGM devices^39^. Furthermore, commercial devices leverage sophisticated calibration algorithms to improve their accuracy. While being outside the scope of this investigation, a calibration algorithm that considers environmental settings, MHD parameters, and previous measurements (glucose trends), could be developed to improve the accuracy of the ISFG measurements.

The different CEG zones (Fig. 3) represent different risk levels. Zone A represents the lowest risk. Measurement datapoints falling within the Zone A impose no risk of adverse clinical outcome for the patient. The risk increases through Zones B, C, D, and E. Zone E is associated with the most severe risk for the patients.

The emergence of solutions for CGM based on the use of interstitial fluid has motivated research on the lag time between blood glucose and glucose in ISF^34^. However, no clear consensus on this topic has yet been reached. The microcirculation in the skin is a complex dynamic phenomenon that causes inter- and intra-subject variations in the lag time^26,28,40^. Furthermore, different ISF reservoirs and different sampling mechanisms can lead to different results. While the clinical relevance of the ISFG has yet to be fully understood, current approaches for CGM in the interstitial fluid using needles have proven to be valuable for people with both type 1 and type 2 diabetes. Our results are consistent with the literature, showing a temporal lag between CBG concentrations and ISFG (Fig. S5). Our results also indicate different lag times among different individuals. For this reason, we calibrated our measurements on ISF for individual experiments (Supplementary Information). Hence, the specific lag times we present should be considered as parameters in an empirical model – rather than direct quantitative and absolute measurements of the time lags. Finally, we note that continuous ISFG and CBG measurements would be better suited to lag quantification. However, the experiment was not designed to target the lag times as that quantification was not a primary goal of this work. Thus, the temporal resolution of our data imposes limitations on the lag assessment and the lag estimates should be treated accordingly. Our future research on a system integrating MHD extraction with electrochemical biosensors will enable a more thorough investigation of the lag time and discussion of its clinical implications.

The determination of glucose concentration in interstitial fluid was done by using a standard fluorometric assay. In this way, the glucose concentration in ISF and blood were measured by two independent methods, in order to minimize the uncertainty related to electrochemical biosensors commonly used in glucometers. In future work, the ISF glucose will be determined by an electrochemical biosensor, which will allow miniaturization of the device. We have demonstrated the integration of MHD and electrochemical sensors for glucose monitoring in a previous work ^41^. As evident from that work, the use of electrochemical sensors overcomes the need for sample collection because the samples can be measured at the extraction site. As the electrochemical sensors are thin (e.g., 200 μm), small magnets can be arranged in close proximity to the skin to generate the desired B-field at the extraction site. For instance, a Neodymium, N52, nickel-plated magnet 5 × 5 × 3 mm (Q-05–05-03-N52N, Supermagnete, Germany) provides a similar magnetic field strength (300 mT) near its surface.

The use of electrochemical sensors also allows reducing the sample dilution. In this study, the interstitial fluid samples were collected and diluted into 200 µL of PBS. Extraction times of 5 min allowed us to collect sufficient volumes of interstitial fluid to enable the quantification of glucose using standard fluorometric assays. Smaller sample volumes and consequently shorter extraction times should be needed when the sample dilution is lower. The extraction time limits the frequency of the measurements. Hence, shorter extraction times are desired. Most CGM devices measure glucose levels at time intervals (e.g., up to every 5 min) rather than continuously. A consequent reduced accuracy during periods of rapidly changing glucose levels has been documented^42^. However, CGM offers the advantage of facilitating frequent glucose measurements for prolonged periods. This gives patients and physicians reliable information about the health condition. In particular, time in range (TIR), enabled by CGM, has demonstrated big value in diabetes diagnosis and treatment^43–45^.

Figure 4 shows the effect of the MHD extraction on skin. The data demonstrated that the skin barrier function was not significantly affected by the extraction when comparing to skin under the control well. We observed an increase in TEWL under each well, including the control (Fig. 4b). Its presence in the control well implies that this increase mostly results from the skin occlusion. The temporary dysfunction of the skin barrier after occlusion may result from increased water content in stratum corneum in the absence of evaporation, leading to temporary disrupted structure of the skin barrier’s lipid multilayer^46^. Related to this disruption, we observed increased epidermal and dermal water content after the extraction experiments (Fig. 4c). This increase was greater in the MHD active sensor locations compared to the control well. One may hypothesize that any active means to percolate fluid flow through the skin towards its surface likely leads to a transient increase in trapped fluid within the skin. The participants were queried about the presence of adverse skin reactions (e.g., redness, rash, itching) on the test day, the next day, one week after the test day, and four weeks after the test day. Of the 5 participants, one reported erythema and absence of other symptoms on the day of the experiment. Another participant reported erythema persisting the day of the experiment and to the next day but, more importantly, none of the participants had any evidence of rash after one week or after four weeks post experiments. Overall, the volunteers considered the experience pleasant. They reported slight tingling sensation in the beginning of the experiment. The sensation was usually present only in the first few seconds of each extraction. Furthermore, in most cases the sensation usually disappeared within the first hours of the experiment. These reports are consistent with the skin property and skin reaction data indicating that the extraction itself is not causing damage to the skin.

## Outlook

We present the first ever study in humans of the safety and effectiveness of MHD to extract dermal ISF to enable needle-free monitoring of glucose. Our results show a high correlation between the concentration of glucose in blood and the concentration of glucose in samples of ISF extracted with MHD. Furthermore, no evidence of long-lasting effects on the skin were observed. Our results indicate a high potential value of MHD as a tool to enable needle-free glucose monitoring. Beyond glucose monitoring, sampling of ISF with MHD has the potential to enable monitoring of other analytes also present in ISF.

## Materials and methods

### Materials

D-Glucose (#G8270-100G) was purchased from VWR, 10 mM PBS powder pouches (#P3813), Glucose oxidase (GOx, #G7141-1MU), Horseradish peroxidase (HRP, #P8250-25KU) from Sigma Aldrich, and AmplexRed (#A12222) from ThermoFisher. All the chemicals were used without additional purification. 75 mM glucose drinks (GlucoTest) were purchased from Suomen diabeteskauppa (Finnish Diabetes Shop, www.diabeteskauppa.fi).

### Extraction of interstitial fluid

Extraction involved applying 300 µA/cm^2^ current density through the skin using a custom-made current source (block diagram in Fig. S1), two Ag/AgCl disk electrodes (r = 2 mm, Warner Instruments) and two neodymium magnets (70 × 70 × 30 mm^3^, Goliath, Supermagnete, Germany). An aluminium spacer maintained 8 cm distance between the magnets. The extracted ISF was collected into circular acrylic wells (fluid contact area to the skin: 0.5 cm^2^) designed for this experiment and ordered from Protohouse (Salo, Finland). These wells were attached to the volunteer’s wrist using double-sided tape (9474LE, 3 M) and filled with 200 µL of PBS. Two wells were used for extraction (distal and central), whereas the third well (proximal) was used as a control well (passive diffusion alone) (Fig. 1a). The control well was used for two purposes. First, to determine the background noise in the sample analysis. Second, as a reference for the skin damage assessment. The distal well was placed 2 cm from the wrist bone (ulna head). The central and proximal wells were placed further along the arm, with 5 mm outer wall-to-wall distance from each other. If the volunteer had any skin damage, such as scars or rash, the wells were placed outside of affected areas. The wells and the skin were rinsed twice with PBS. Rinsing was followed by filling the wells with 200 µL of PBS and then starting the extraction. Two Ag/AgCl disk electrodes (r = 2 mm, E202, Warner Instruments) inserted into the wells ensured a low-impedance electrical connection with the solution and prevented pH changes due to the electric current. The electrodes were positioned carefully into the PBS solution while avoiding touching the skin. Both the current intensity and the voltage between the extraction wells was monitored using a DMM (34465A, Keysight).

The two permanent magnets created a magnetic field along the lateral axis of the volunteer’s arm (Fig. 1c). At the extraction site, the measured magnetic field was 300 mT. The sampling of ISF with MHD was induced by driving an electric current (300 µA/cm^2^) through the electrodes, the electrolyte in the extraction wells, and the skin in the magnetic field (Fig. 1d and Fig. S1). The current (***J***) was driven orthogonally to the magnetic field (***B***) to create a Lorenz force (***F***) pointing from the dermis towards the skin surface, thus inducing magnetohydrodynamic extraction of ISF. The first extraction was always conducted with the distal well working as the cathode. Each extraction was followed by a 5 min collection time, where samples from each well were transferred into microtubes. The wells were then rinsed with PBS and fresh PBS added for the subsequent extraction. The polarity of the current and the direction of the magnetic field were swapped for each subsequent extraction. Reference blood glucose concentrations of the volunteer were measured using a glucometer (Contour XT, Ascensia Diabetes Care) following the manufacturer’s instructions. The CBG samples were taken from the arm which was not used for MHD extraction. This meant the right arm for the first five experiments and the left arm for the last five.

### Glucose tolerance test

All participants for the study were volunteers who had submitted informed consent in writing before the tests. The experimental methodology complies with the clinical pilot study plan reviewed and approved by the Helsinki and Uusimaa Hospital District Ethical Committee II. This study is a clinical medical device study according to The Finnish Act on Medical Devices and Equipment (Laki Terveydenhuollon Laitteista ja Tarvikkeista 629/2010) and was performed in accordance with the guidelines within*.* This study was performed in accordance with the Declaration of Helsinki. The volunteer entered the test after fasting for 9 h. The test started with a first CBG measurement followed by an extraction of ISF. After 120 min, a second CBG measurement was taken. Then, the volunteer consumed a glucose drink (75 g of glucose, GlucoTest). Afterwards, 8 more CBG measurements were taken, one every 20 min (total of 10 during the test). The MHD extractions and CBG measurements were performed simultaneously over the duration of the experiment. MHD and CBG glucose readings were taken from both the left (n = 5) and the right (n = 5) arms. In total, 10 tests were performed on five different volunteers (2 males and 3 females, age range 28–43 years). Each volunteer participated twice in different days. First experiment was conducted from the left arm and the second from the right arm.

### Skin parameter measurements

Skin was washed with soap (Neutral ®), rinsed with water, and dried with a paper towel both before and after the extraction experiment. After the wash, TEWL and skin moisture were measured at each extraction site and at the diffusion control site. The time between washing the arm and skin measurements was kept constant (15 min). Measurements were performed using VapoMeter^47,48^, and MoistureMeterD^49^ Compact (Delfin Technologies, Finland) according to the manufacturer’s instructions. Each location was measured three times, and an average of the three measurements was used in the analysis.

### Determination of glucose concentration in the extracted ISF samples

The glucose concentration in the extracted ISF samples was measured using a GOx/HRP/AmplexRed assay. First, 20 µL of standard glucose solutions and each extracted sample were individually pipetted in a 384-well plate in duplicate (black, Corning). Next, we added 10 µL of 250 µM AmplexRed reagent. After adding the reagent, the plate was transferred to a plate reader (Varioskan LUX, Thermo Fisher), which automatically injected 10 µL of GOx/HRP solution to each well to initiate a cascade of reaction: ${C}_{6}{H}_{12}{O}_{6}+{O}_{2}\stackrel{GOx}{\to }{C}_{6}{H}_{10}{O}_{6}+{H}_{2}{O}_{2}; {H}_{2}{O}_{2}+ \mathrm{AmplexRed}\stackrel{HRP}{\to } resorfurin+ {H}_{2}O$

Resorufin emits a fluorescent signal at 585 nm upon its excitation with λ = 571 nm light^50^. The reaction was allowed to progress for 8 min, and the end point value was used to quantify the glucose concentration in each sample by using a standard calibration curve obtained from the standard glucose samples.

Samples from the control well were subtracted from each corresponding MHD cathode well sample. Subtracting the control sample readings serves to remove the contribution of glucose due to passive diffusion from the total glucose in our samples. The distal and central well MHD cathode samples for each sample pair were averaged. Next, least squares fits to the control-subtracted MHD cathode samples vs. CBG concentrations were made for each individual experiment. The MHD control-subtracted cathode samples for each experiment were then divided by their respective slopes from the least squares fits. This approach enables a robust analysis of correlation with minimal data processing. The corresponding Consensus Error Grid is shown in Fig. 3. It is important to note that this calibration is done retrospectively, using all the data from each individual experiment, as opposed to in real-time. We acknowledge the importance of a real-time algorithm. For these data, a real-time algorithm could rely on two CBG reference points: one at the start, and then another near the glucose peak. These points, along with their corresponding ISFG measurements would yield the initial ISFG, as well as scale the ISFG to match the CBG range. However, the lag complicates this calibration. Consequently, alternative calibration methods and improved understanding of the lag via additional clinical studies are desirable. With this in mind, the data presented here are suboptimal for the development of such an algorithm.

The temporal lag between the CBG and ISFG concentrations was considered prior to the calibration. A detailed description of this adjustment and the lag characterization is given in the Supplementary Information (Fig. S5).

The resulting predicted MHD glucose concentrations and CBG reference data were then averaged for each sample over all experiments (i.e. Sample n from all experiments 1–10 were averaged, repeated for each Sample until Sample N). There was one exception for the first experiment, which used 10 min instead of 5 min extractions for the first 4 extractions during the initial warm-up period. Thus, the warm-up period for that experiment was longer than for the other experiments. Consequently, there were two fewer samples in this first experiment compared to the other experiments.

### Data processing

We collected data from the MHD glucose concentrations, CBG reference measurements, skin moisture measurements, and extraction voltages. This pre-processing was performed in Python using standard data-handling libraries (Numpy^51^, SciPy^52^, Pandas^53^, Statsmodels^54^, and Plotly^55^ for display).

The MHD glucose and reference CBG concentrations were measured at different times (see experimental protocol in Fig. 2a). Thus, to compare the two, the reference CBG concentrations were matched in time to the MHD glucose concentrations via linear interpolation. (i.e. a line is fit between two CBG measurements. The point on that line used as a reference CBG is the point that corresponds to the MHD sample time that is being compared. The initial relative MHD and CBG measurement timelines are consistent with the example shown in Fig. 2b and the temporally aligned MHD and CBG glucose concentrations are shown in Fig. 2c).

The times assumed for each MHD glucose concentration sample in the analysis were their collection times (within 1 min of the current being shut off after each extraction). The final MHD concentrations used in our analysis were the mean concentrations when combining the distal and central wells (and thus the sample times for the two wells were averaged for the final analysis). Note that the MHD glucose concentrations were diluted in 200 µL of PBS – thus they are dissimilar from the ISFG concentration, but rather correlate with it. Our calibration compensates for this dilution.

Extraction voltages (Fig. S4) were used to determine the warm-up time by assessing how they drifted on the y-axis over time.

To assess covariance, we performed an ANOVA^56^ and Tukey’s Honestly Significant Difference test^57^. Our models included arm (categorical variable, left or right), the five individual participants (categorical variable, grouped as two experiment days per participant), the ten individual experiments explored individually, regardless of repeat participants (categorical variable), the standard error of the control well (over each experiment duration), and time (continuous variable, in seconds).

We set *p* < 0.05 as the criteria to reject the null hypothesis that the predicted ISFG is equivalent across the different categories in our model. The ANOVA demonstrated that the right and left arms produce equivalent results. When adding participant as a model parameter, the null hypothesis was rejected. To identify the reason, we performed a Tukey’s HSD test and found that participant 2 was an exceptional case. A subsequent Tukey’s HSD test of participant 2, revealed that the experiment #2b (see Fig. 2) was exceptional. This exception is most likely due to an outlier in the ISFG between 150 and 200 min (Fig. 2c). Finally, the ANOVA indicates some change in correlation between predicted ISFG and reference CBG over time (*p* = 0.008). Results from this analysis are summarized in Table 1.

**Table 1:** Model included in the ANOVA analysis. Asterisks indicate parameters having *p* < 0.05, resulting in rejection of the null hypothesis.

Model 1	*p*-value < 0.05
Arm (left or right)	
Participant (5 total)	*
Standard error (control well)	
Time	*

## Supplementary Information

Supplementary Information.

## Data Availability

The datasets generated during and/or analysed during the current study are available from the corresponding author on reasonable request.

## Code availability

Code used for this analysis is available from the corresponding author on reasonable request.

## References

[1] Didyuk O, Econom N, Guardia A, Livingston K, Klueh U (2021). Continuous glucose monitoring devices: Past, present, and future focus on the history and evolution of technological innovation. J. Diabetes Sci. Technol..

[2] Bidonde, J., Fagerlund, B. C., Frønsdal, K. B., Lund, U. H. & Robberstad, B. FreeStyle libre flash glucose self-monitoring system: A single-technology assessment. *Report from the Norwegian Institute of Public Health.* **7**. https://www.ncbi.nlm.nih.gov/books/NBK482068/ (2017). 29553668

[3] Akturk HK, Dowd R, Shankar K, Derdzinski M (2021). Real-world evidence and glycemic improvement using dexcom G6 features. Diabet. Technol. Ther..

[4] Deiss D (2019). Clinical practice recommendations on the routine use of eversense, the first long-term implantable continuous glucose monitoring system. Diabetes Technol. Ther..

[5] Polonsky WH (2017). The impact of continuous glucose monitoring on markers of quality of life in adults with type 1 diabetes: Further findings from the DIAMOND randomized clinical trial. Diabetes Care.

[6] Fokkert M (2019). Improved well-being and decreased disease burden after 1-year use of flash glucose monitoring (FLARE-NL4). BMJ Open Diabetes Res. Care.

[7] Charleer S (2020). Quality of life and glucose control after 1 year of nationwide reimbursement of intermittently scanned continuous glucose monitoring in adults living with type 1 diabetes (FUTURE): A prospective observational real-world cohort study. Diabetes Care.

[8] Šoupal J (2020). Glycemic outcomes in adults with T1D are impacted more by continuous glucose monitoring than by insulin delivery method: 3 years of follow-up from the COMISAIR study. Diabetes Care.

[9] Šoupal J (2016). Comparison of different treatment modalities for type 1 diabetes, including sensor-augmented insulin regimens, in 52 weeks of follow-up: A COMISAIR study. Diabetes Technol. Ther..

[10] Haak T (2017). Use of flash glucose-sensing technology for 12 months as a replacement for blood glucose monitoring in insulin-treated type 2 diabetes. Diabetes Ther..

[11] Ruedy KJ, Parkin CG, Riddlesworth TD, Graham C (2017). Continuous glucose monitoring in older adults with type 1 and type 2 diabetes using multiple daily injections of insulin: Results from the DIAMOND trial. J. Diabetes Sci. Technol..

[12] Mikael Flockhart A (2021). Excessive exercise training causes mitochondrial functional impairment and decreases glucose tolerance in healthy volunteers. Cell Metab..

[13] Scott S (2021). Post-exercise recovery for the endurance athlete with type 1 diabetes: A consensus statement the importance of protein after exhaustive endurance exercise View project Post-exercise recovery for the endurance athlete with type 1 diabetes: a consensus statement. Lancet Diabetes Endocrinol..

[14] Scallan, J., Huxley, V. H. & Korthuis, R. J. Capillary fluid exchange: Regulation, functions, and pathology. *Integrated Systems Physiology: From Molecule to Function to Disease. ***2**, 1–94 10.4199/C00006ED1V01Y201002ISP003 (Morgan & Claypool Life Sciences, 2010). 21452435

[15] Sansalone, V., Kaiser, J., Naili, S. & Lemaire, T. Interstitial fluid flow within bone canaliculi and electro-chemo-mechanical features of the canalicular milieu. *Biomech Model in Mechanobiol. 2012 12:3* **12**, 533–553 10.1007/s10237-012-0422-7 (2012). 10.1007/s10237-012-0422-7 22869342

[16] Cengiz E, Tamborlane WV (2009). A tale of two compartments: Interstitial versus blood glucose monitoring. Diabetes Technol Ther..

[17] Dye L (2010). Correspondence of continuous interstitial glucose measurement against arterialised and capillary glucose following an oral glucose tolerance test in healthy volunteers. Br. J. Nutr..

[18] Rao G, Glikfeld P, Guy RH (1993). Reverse Iontophoresis: Development of a noninvasive approach for glucose monitoring. Pharml. Re..

[19] Pu Z (2021). A thermal activated and differential self-calibrated flexible epidermal biomicrofluidic device for wearable accurate blood glucose monitoring. Sci Adv..

[20] Leboulanger B, Guy RH, Delgado-Charro MB (2004). Reverse iontophoresis for non-invasive transdermal monitoring. Physiol. Meas..

[21] Mitragotri S, Coleman M, Kost J, Langer R (2000). Transdermal extraction of analytes using low-frequency ultrasound. Pharm. Res..

[22] Mitragotri S, Coleman M, Kost J, Langer R (2000). Analysis of ultrasonically extracted interstitial fluid as a predictor of blood glucose levels. J. Appl. Physiol..

[23] Diessel E (2016). Nanoliter serum sample analysis by mid-infrared spectroscopy for minimally invasive blood-glucose monitoring. Appl. Spectrosc..

[24] Hanna J (2020). Noninvasive, wearable, and tunable electromagnetic multisensing system for continuous glucose monitoring, mimicking vasculature anatomy. Sci. Adv..

[25] Zierler K (1999). Whole body glucose metabolism. Am. J. Physiol..

[26] Scuffi C, Lucarelli F, Valgimigli F, Diagnostics AM (2012). Minimizing the impact of time lag variability on accuracy evaluation of continuous glucose monitoring systems. J. Diabetes Sci. Technol..

[27] Davey RJ, Low C, Jones TW, Fournier PA (2010). Contribution of an intrinsic lag of continuous glucose monitoring systems to differences in measured and actual glucose concentrations changing at variable rates in vitro. J. Diabetes Sci. Technol..

[28] Basu A (2013). Time lag of glucose from intravascular to interstitial compartment in humans. Diabetes.

[29] Reiterer F (2017). Significance and reliability of MARD for the accuracy of CGM systems. J. Diabetes Sci. Technol..

[30] Parkes JL, Slatin SL, Pardo S, Ginsberg BH (2000). A new consensus error grid to evaluate the clinical significance of inaccuracies in the measurement of blood glucose. Diabetes Care.

[31] ISO - ISO 15197:2013 - In vitro diagnostic test systems — Requirements for blood-glucose monitoring systems for self-testing in managing diabetes mellitus. https://www.iso.org/standard/54976.html.

[32] Hakala TA (2021). Sampling of fluid through skin with magnetohydrodynamics for noninvasive glucose monitoring. Sci. Rep..

[33] Wientjes KJC, Schoonen AJM (2018). Determination of time delay between blood and interstitial adipose tissue glucose concentration change by microdialysis in healthy volunteer. Int. J. Artif. Organs..

[34] Boyne MS, Silver DM, Kaplan J, Saudek CD (2003). Timing of changes in interstitial and venous blood glucose measured with a continuous subcutaneous glucose sensor. Diabetes.

[35] Akdeniz M, Gabriel S, Lichterfeld-Kottner A, Blume-Peytavi U, Kottner J (2018). Transepidermal water loss in healthy adults: A systematic review and meta-analysis update. Br. J. Dermatol..

[36] Pinto PC, Rodrigues LM (2005). Influence of the time of occlusion on the quantitative parameters obtained by modelling trans-epidermal water loss curves to describe the human cutaneous barrier functionin vivo. Med. Biol. Eng. Comput..

[37] Garg SK (2022). Accuracy and safety of Dexcom G7 continuous glucose monitoring in adults with diabetes. Diabetes Technol. Ther..

[38] Engler R, Routh TL, Lucisano JY (2018). Adoption barriers for continuous glucose monitoring and their potential reduction with a fully implanted system: Results from patient preference surveys. Clin. Diabetes..

[39] Heinemann L (2020). Benefits and limitations of MARD as a performance parameter for continuous glucose monitoring in the interstitial space. J. Diabetes Sci. Technol..

[40] Kovatchev BP, Shields D, Breton M (2009). Graphical and numerical evaluation of continuous glucose sensing time lag. Diabetes Technol. Ther..

[41] Kemp E (2022). Influence of enzyme immobilization and skin-sensor interface on non-invasive glucose determination from interstitial fluid obtained by magnetohydrodynamic extraction. Biosens. Bioelectron..

[42] Pleus S (2015). Rate-of-change dependence of the performance of two CGM systems during induced glucose swings. J. Diabetes Sci. Technol..

[43] Wright EE, Morgan K, Fu DK, Wilkins N, Guffey WJ (2020). Time in range: How to measure it, How to report it, and its practical application in clinical decision-making. Clin Diabetes..

[44] Beck RW (2019). Validation of time in range as an outcome measure for diabetes clinical trials. Diabetes Care.

[45] Ceriello A (2022). Glycaemic management in diabetes: Old and new approaches. Lancet Diabetes Endocrinol..

[46] Zhai H, Maibach HI (2002). Occlusion vs skin barrier function. Skin Res. Techol..

[47] Steiner M, Aikman-Green S, Prescott GJ, Dick FD (2011). Side-by-side comparison of an open-chamber (TM 300) and a closed-chamber (Vapometer^TM^) transepidermal water loss meter. Skin Res. Technol..

[48] de Paepe K, Houben E, Adam R, Wiesemann F, Rogiers V (2005). Validation of the VapoMeter, a closed unventilated chamber system to assess transepidermal water loss vs the open chamber Tewameter. Skin Res. Technol..

[49] Mayrovitz, H. N. Assessing free and bound water in skin at 300 MHz using tissue dielectric constant measurements with the MoistureMeterD. *Lymphedma.*10.1007/978-3-319-14493-1_13 (2015).

[50] Debski D (2016). Mechanism of oxidative conversion of Amplex® Red to resorufin: pulse radiolysis and enzymatic studies. Free Radic. Biol. Med..

[51] Harris CR (2020). Array programming with NumPy. Nature.

[52] Virtanen P (2020). SciPy 1.0: Fundamental algorithms for scientific computing in Python. Nat. Methods.

[53] McKinney, W. Data structures for statistical computing in python. *Proceedings of the 9th Python in Science Conference* 56–61 10.25080/MAJORA-92BF1922-00A (2010).

[54] Seabold, S. & Perktold, J. Statsmodels: Econometric and statistical modeling with python. *Proceedings of the 9th Python in Science Conference* 92–96 10.25080/MAJORA-92BF1922-011 (2010).

[55] Plotly Technologies Inc. Collaborative data science. *Plotly Technologies Inc.* https://plotly.com/chart-studio-help/citations/ (2015).

[56] Girden, E. *ANOVA: Repeated measures*. (1992).

[57] Tukey, J. *Exploratory data analysis*. Addison-Wesley (1977).

