# Supplementary material for: Pilot study in human healthy volunteers on the use of magnetohydrodynamics in needle-free continuous glucose monitoring
Source: Sci Rep. 2022 Nov 9;12:18318. doi: 10.1038/s41598-022-21424-9 (PMC9646842; doi:10.1038/s41598-022-21424-9)
Supplement: Supplementary file 1 — Supplementary Information. [file 41598_2022_21424_MOESM1_ESM.docx]

**SUPPLEMENTARY INFORMATION**

Pilot study in human healthy volunteers on the use of magnetohydrodynamics in needle-free continuous glucose monitoring

Tuuli A. Hakala^1,#^, Laura K. Zschaechner^1,5,#^, Risto T. Vänskä^1,5^, Teemu A. Nurminen^1^, Melissa Wardale^1^ , Jonathan Morina^1^, Zhanna A. Boeva^1,2^, Reeta Saukkonen^1^ ,Juha-Matti Alakoskela^1,3^, Kim Pettersson-Fernholm^1,4^, Edward Hæggström ^1,5^, and Johan Bobacka ^1,2,^ , Alejandro García Pérez^1,^ *

^#^ These authors contributed equally to the work

^1^ *Glucomodicum Ltd, A.I. Virtasen Aukio 1, 00560 Helsinki, Finland*

*^2^Laboratory of Molecular Science and Engineering, Faculty of Science and Engineering, Åbo Akademi University, Henriksgatan 3 , 20500 Turku/Åbo, Finland*

*^3^Skin and Allergy Hospital, Meilahdentie 2, 00250 Helsinki, Finland*

^4^ *Nefrologian poliklinikka, Helsinki University Hospital, Haartmaninkatu 4, 00029 Helsinki, Finland*

^5^ *Department of physics, University of Helsinki,* *Gustaf Hällströmin katu 2, 00560 Helsinki, Finland*

**ag@glucomodicum.com*

**Table of Contents:**

**Supplementary Figure 1:** Block diagram of the electronics set up

**Supplementary Figure 2:** Extraction time optimization

**Supplementary Figure 3:** Extraction voltages during glucose tolerance test

**Supplementary Figure 4:** Raw TEWL and MoistureMeterD readings

**Supplementary Figure 5:** Characterization of the ISFG and CBG temporal lag

**
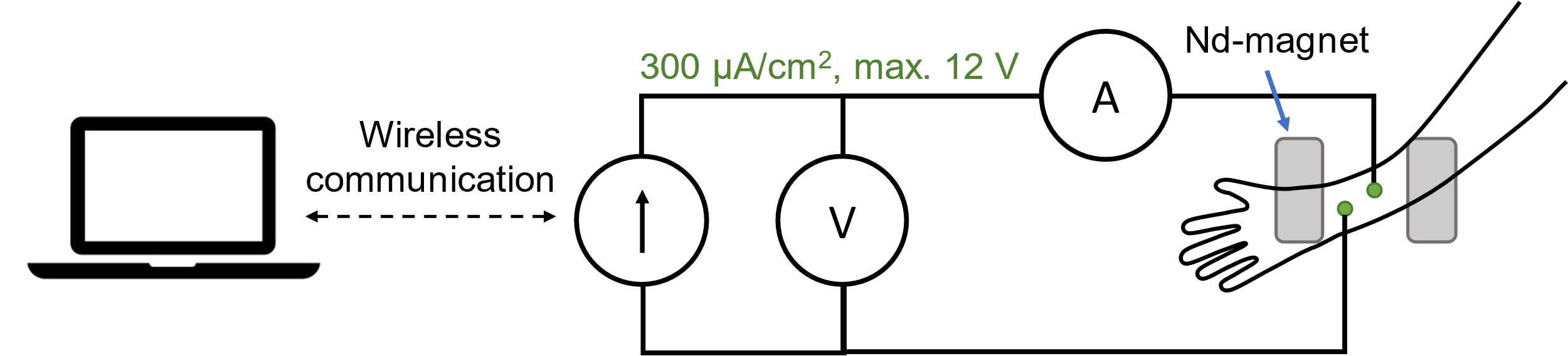
Figure S1 Block diagram of the electronics set up** Battery-powered constant current source is used to apply a current density of 300 μA/cm^2^ through the skin via acrylic wells attached to skin surface. The current source is controlled wirelessly via Bluetooth from a computer. Output current and voltage between the wells is monitored using a commercial multimeter.


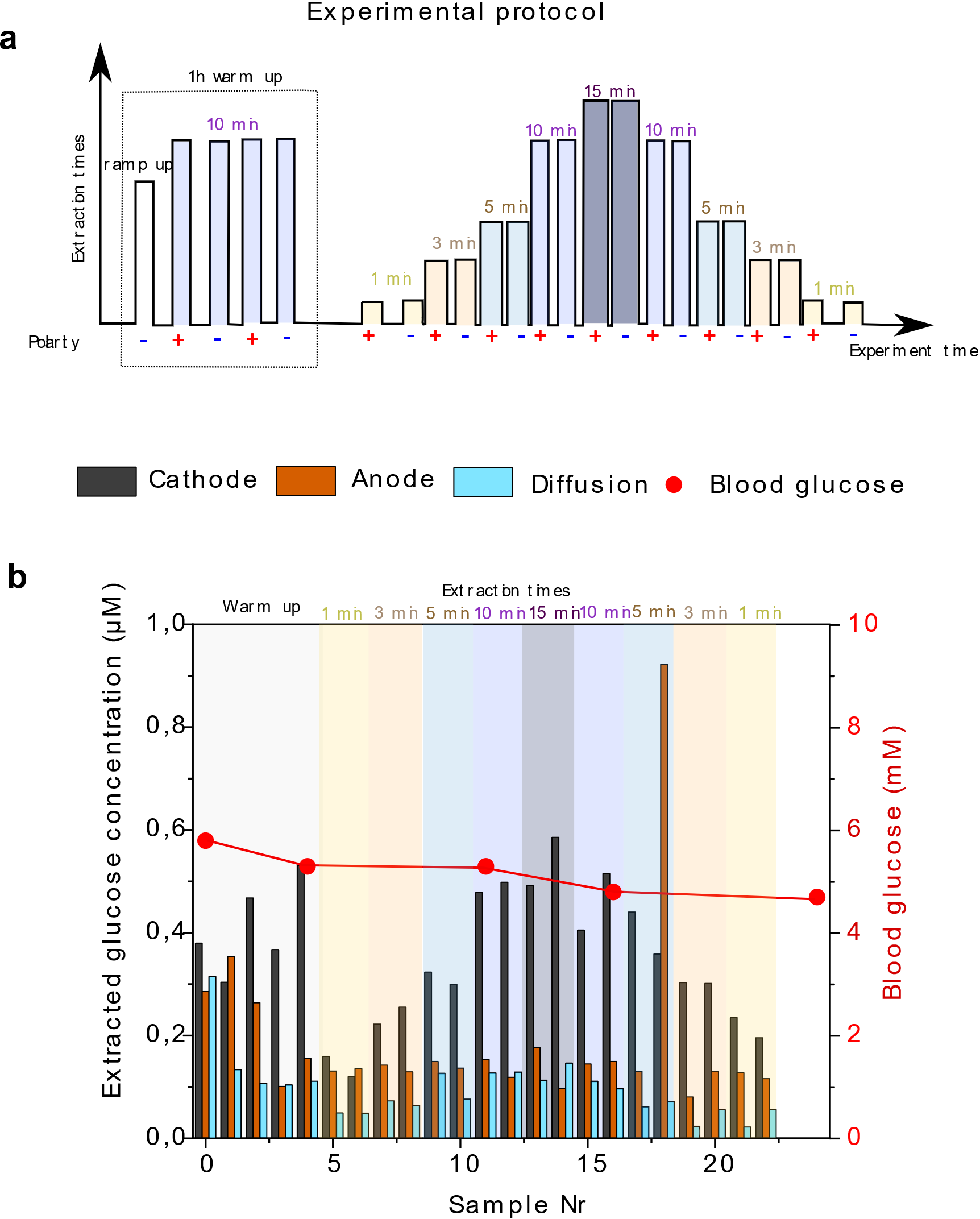


**Figure S2 Extraction time optimization.** **a** Scheme for experiment protocol. Experiment started with warm up that consists of 7 min ramp up and four 10 min warm up extractions. After the warm-up the extraction times were increased from 1 to 15 min always measuring di direct (+) and inversed (-) polarity after each other. After the two 15 min extractions the extraction times were decreased starting from 10 min to 1 min. **b** Raw data plotted form a representative experiment where cathode samples are black bars, anode samples are orange bars and diffusion samples as light blue bars. The glucose concentration after each extraction was measured spectrophotometrically using AmplexRed assay. Blood glucose of the volunteer was also measured using glucose meter and represented as red dots in the graph.

**Optimization of extraction times.** We started the experiments by screening different extraction times. The volunteers were advised to fast 9 hours before the experiment to minimize the changes of blood glucose during the experiment. The experiment took 5 hours and started with 1 hour warm-up consisting of 5 extraction cycles (Figure S3**a**). The first extraction cycle was performed by ramping up the current to 300 µA/cm^2^ using 50 µA increments (15 s each). After reaching 300 µA/cm^2^ the current was kept on for 7 minutes. This was followed by four 10 min extractions which were considered as “warm-up”. After each extraction PBS was collected from the wells and transferred to a 500µl tube, and each well was rinsed with fresh PBS. The current polarity was changed between each extraction. After the warm-up, the extraction cycles were performed for 1 min, 3 min, 5 min, 10 min and 15 min in ascending order first followed by descending order. The extraction cycles were conducted as pairs so that both current directions (direct and reverse) were performed. This meant that in the first extraction an electrode in a distal well was used as a cathode and the one in the central well was used as an anode. In the second extraction, the electrode in the distal well was used as the anode, and one in the central well was used as a cathode. This way, current flow in two opposite directions was applied to the extraction cell. Raw data from a single volunteer is shown in figure S3**b.** The data shows that by increasing the extraction time, the amount of extracted glucose is elevated in the cathode sample. However, the trend is not seen in the anode or diffusion samples. Furthermore, the blood glucose values measured during the experiment (red dots, Fig S3**b**), remained relatively stable, confirming that the changes seen in the cathode are not due to changes in blood glucose. Our criteria for choosing an extraction time were to minimize both the extraction time and uncertainty. Therefore, 5 min extraction time was selected.


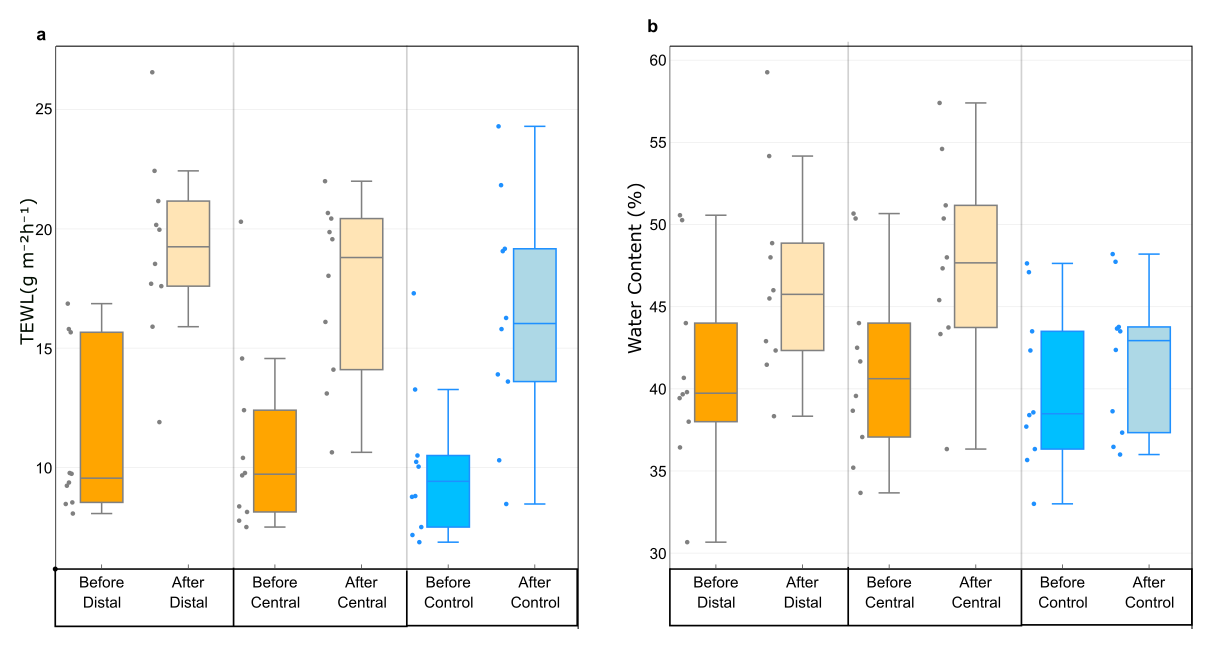


**Figure S3. Skin properties.** TEWL and skin water content readings were performed before and after the extraction experiment. The individual differences in both readings were found to be large, thus we took the delta values (after-before) for each volunteer and presented them in the main paper (Figure 4**b**)


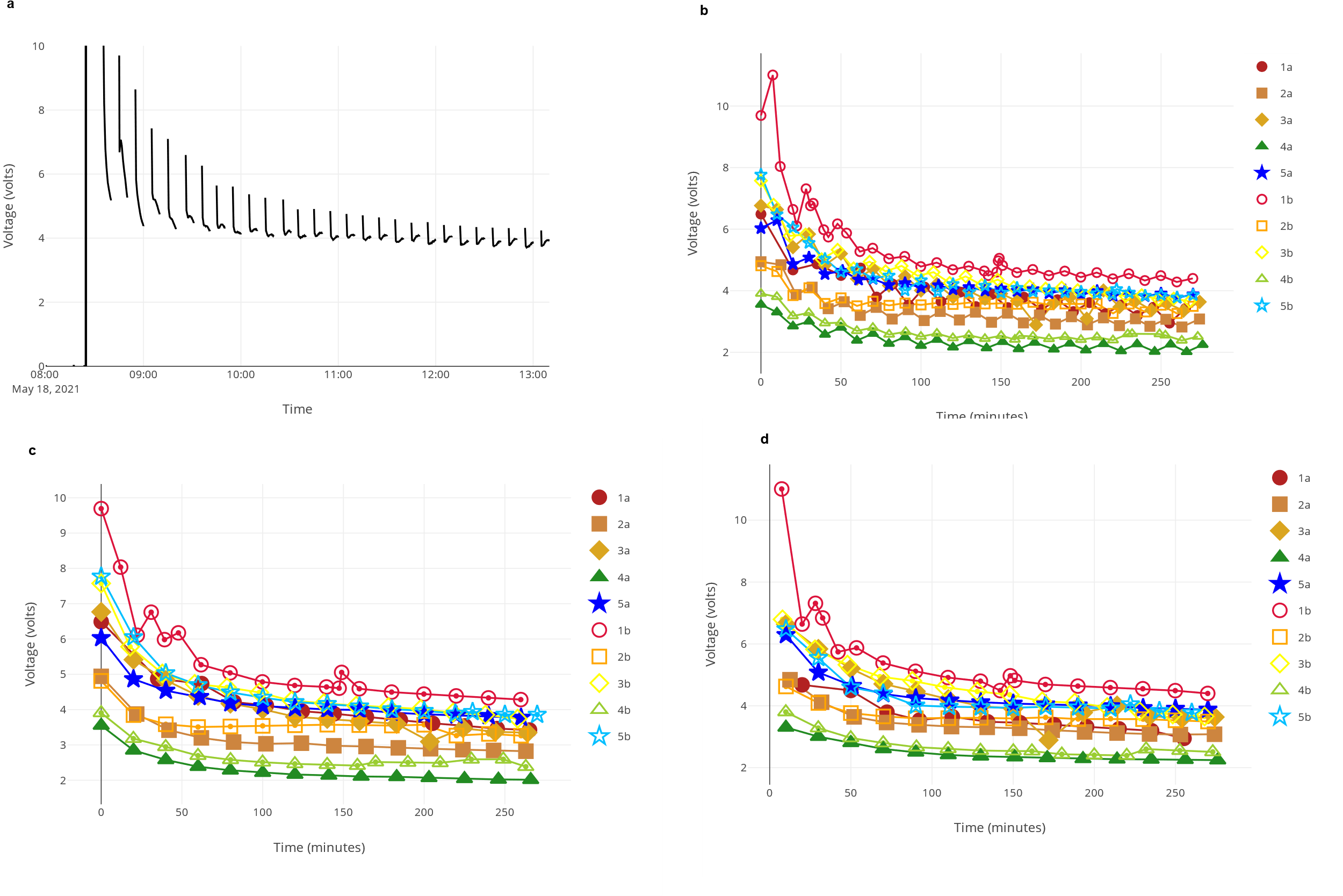


**Figure S4 a** Representative raw extraction voltage data from a glucose tolerance test. The data shown in this panel correspond to experiment 5a**. b** Average extraction voltages (removing the first 20% and final 10% from each signal to isolate the stabilized/flat part) from all 10 glucose tolerance tests. Current source with a compliance voltage of 12 V was used to limit the voltage for safety reasons. The voltage associated with each signal is that of the mean of its stabilized part. Consequently, the times assumed for the extraction voltages correspond to the mean time of the stabilized range of each signal. The corresponding MHD samples used in the analysis are those immediately following each extraction. The average voltage data are separated into the direction of current **c** direction one (distal well as cathode and **d** direction 2 (central well as cathode).

**The temporal lag between ISFG and CBG.** To further understand the effect of the temporal lag, we performed additional analysis and comparison between different lag models. First, we applied a single uniform lag across all experiments. We introduced this lag as a relative shift between the MHD concentrations and CBG prior to applying the least-squares calibration.

To determine the optimal lag across all experiments, we explored uniform lags. The lag magnitudes were tested at 5-minute intervals (*e.g.* lag = 0 minutes, lag = 5 minutes, *etc*.) in order to avoid going beyond the temporal resolution of our data. The tested lags for the uniform lag applied across all experiments ranged from 0-30 minutes. While testing these lags, we defined numerical optimization criteria. These criteria were used to evaluate the correlation between the CBG concentrations and the ISFG concentrations resulting from each lag in our testing range (the relation shown in Fig 3c, but for lags ranging from 0-30 minutes). The correlation was found using ordinary least squares fit to the ISFG and CBG concentrations. The principles behind the numerical optimization criteria are: 1) the slope should be as close to unity as possible, 2) the intercept should be close to zero, and 3) R^2^ should be close to unity. The former two criteria aim for a match between ISFG and CBG concentrations. The latter criterium assesses the strength of the correlation between ISFG and CBG. These criteria yielded an optimal lag of 15 minutes. The tested uniform lags, their fit criteria, and the resulting MARD and PARD values are shown in Table S**1.**

The uncertainties in the uniform optimal lag were also determined using the slope, intercept, and R^2^. The uncertainties encompass lags that: 1) have a slope greater than 0.9, but less than 1.1, 2) have an intercept (absolute value) corresponding to less than 0.4 mM, and 3) have an R^2^ greater than 0.9. Using this procedure and these criteria, we determined an optimal uniform lag of 15 minutes (Table S**1**, Fig S**5**).

**Table S1:** Constant, uniform lags (same across all experiments) presented at 5-minute intervals. The lag optimization criteria (slope, intercept, and R^2^) are included, as well as the resulting MARD and PARD.

| **Lag (minutes)​** | **Slope​** | **Intercept​ (mM)** | **R^2^​** | **MARD** | **PARD** |
| --- | --- | --- | --- | --- | --- |
| **0** | 0.87 | 1.06 | 0.71 | 16.9% | 16.7% |
| **5** | 0.93 | 0.57 | 0.84 | 15.9% | 15.8% |
| **10** | 0.96 | 0.31 | 0.92 | 15.1% | 15.1% |
| **15** | 0.96 | 0.34 | 0.94 | 14.6% | 14.6% |
| **20** | 0.93 | 0.53 | 0.88 | 15.3% | 15.4% |
| **25** | 0.92 | 0.59 | 0.91 | 15.0% | 15.0% |
| **30** | 0.89 | 0.84 | 0.89 | 15.1% | 15.0% |


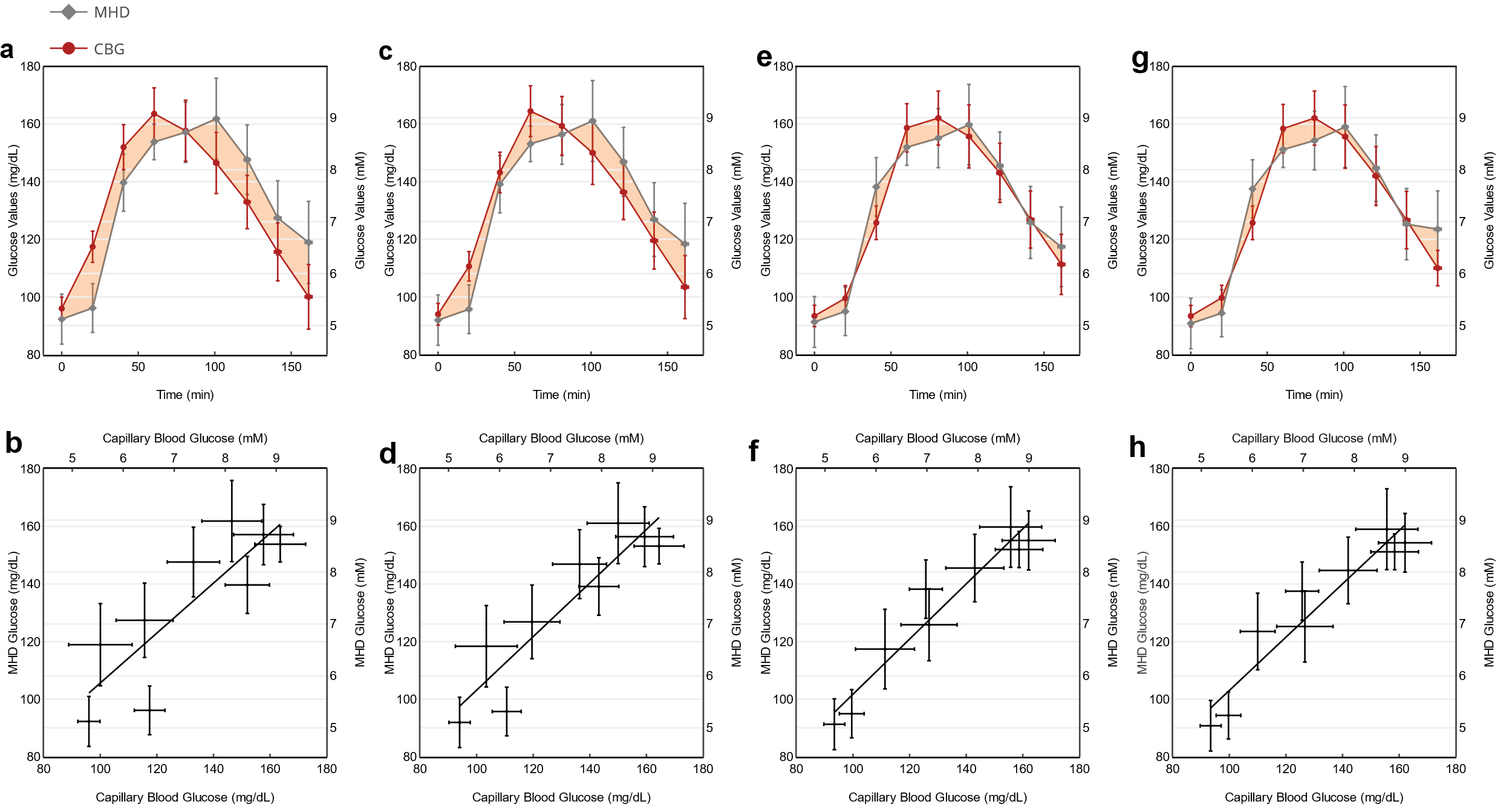


**Figure S5**. **Investigation of the uniform lag time for the average of all 10 experiments.** Top row: The MHD glucose (grey) and reference blood glucose (red) measurements for a range of temporal lag concentrations. The shaded areas are representative of how much the MHD glucose differs from the reference blood glucose for each lag. The starting time-zero is the time corresponding to these first sample used for analysis. Bottom row: The same data, but with MHD glucose (y-axis) plotted against the reference blood glucose (x-axis). Lag times are: **a** and **b** 0 min, **c** and **d** 5 min, **e** and **f** 15 min, and **g** and **h** 25 min. Error bars are the standard deviation of the mean for each averaged sample. They are representative of the spread in glucose values across experiments.

Since lags are known to differ between individuals and over time, we also optimized the lags for each experiment individually using the slope, intercept, and R^2^. These criteria, however, were relaxed for the individual lags as the data from individual experiments presented a lower signal to noise ratio than when all 10 experiments are averaged together. The slope and intercept criteria were modified to reflect this change (multiplied by $\surd10$). These modified criteria are: 1) a slope between 0.7, and 1.3, 2) an intercept (absolute value) corresponding to less than 1.3 mM, and 3) an R^2^ greater than 0.5 (minimum threshold for a moderate correlation). This calibration using lags optimized for each individual experiment is what is quoted as the main result for this paper. The optimized individual lags ranged from 10 to 40 minutes (mean ~ 20 minutes). These data, including the temporal lags between interstitial fluid and blood glucose are shown in Figure 2**b** and Figure 2**c**.


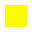


Regarding the individually optimized lags, experiments 2b, 3a, 3b, 4b, and 5a did not meet the optimization criteria. These experiments were thus assigned optimal lag value determined by averaging all experiments together (i.e. 15 min). Experiment 2a nearly met the optimization criteria with a reasonably high R^2^ of > 0.8 The corresponding R^2^ for experiment 2a when using the default 15-minute lag was 0.7. Thus, in our final analysis we used the best lag obtained via fitting for experiment 2a. We include the individually optimized lags quality of fit parameters in Table S**2**.

**Table S2:** Individually optimized lags, their uncertainties, and parameters related to the lag optimization criteria (slope, intercept, and R^2^).

| **Experiment** | **Lag (minutes)** | **slope** | **Intercept (mM)** | **R^2^** |
| --- | --- | --- | --- | --- |
| 1 a | 10 ± 5 | 1.07 | -0.53 | 0.84 |
| 1 b | 10 ± 5 | 0.84 | 1.17 | 0.88 |
| 2 a | 30 ± 10 | 1.38 | -3.77 | 0.83 |
| 2 b | 15 | n/a | n/a | n/a |
| 3 a | 15 | n/a | n/a | n/a |
| 3 b | 15 | n/a | n/a | n/a |
| 4 a | 40 ± 10 | 1.17 | -1.14 | 0.43 |
| 4 b | 15 | n/a | n/a | n/a |
| 5 a | 15 | n/a | n/a | n/a |
| 5 b | 20 | 1.21 | -1.45 | 0.84 |

**Table S3:** Data and lag properties for 1) the uniform 15-minute lag applied to all experiments and 2) the individually optimized lags.

| **Lag properties** | **Slope​** | **Intercept (mM)​** | **R^2^​** | **MARD** | **PARD** |
| --- | --- | --- | --- | --- | --- |
| uniform lag (15 minutes) | 0.96 | 0.34 | 0.94 | 14.6% | 14.6% |
| Individually optimized lags (Table S**2**) | 0.97 | 0.24 | 0.92 | 12.9% | 13.1% |
